# Evaluation of alveolar bone hypomineralization in pediatric hypophosphatasia using orthopantomography

**DOI:** 10.1038/s41598-022-05171-5

**Published:** 2022-01-24

**Authors:** Rena Okawa, Takashi Nakamoto, Saaya Matayoshi, Kazuhiko Nakano, Naoya Kakimoto

**Affiliations:** 1 Department of Pediatric Dentistry, Osaka University Graduate School of Dentistry, 1-8 Yamada-oka, Suita, Osaka 565-0871 Japan; 2 Department of Oral and Maxillofacial Radiology, Graduate School of Biomedical and Health Sciences, Hiroshima University, 1-2-3 Kasumi, Minami-ku, Hiroshima 734-8553 Japan

**Keywords:** Dental diseases, Metabolic disorders, Oral diseases, Paediatric research, Oral manifestations

## Abstract

Hypophosphatasia (HPP) is a metabolic disease characterized by impaired bone mineralization and early exfoliation of primary teeth. This study was performed to develop a method for quantitatively evaluating alveolar bone hypomineralization using orthopantomographic images. Alveolar bone density was defined according to the pixel values and corrected by brightness shown by an indicator applied to the orthopantomographic device. Images of 200 healthy subjects (aged 2–15 years) were classified into five age groups. The corrected pixel values were significantly lower in the younger group than in those aged 14–15 years (2–4, 5–7, and 8–10 years versus 14–15 years: *P* < 0.0001, 11–13 years versus 14–15 years: *P* < 0.01). Orthopantomographic images of 17 patients with HPP were evaluated. The corrected pixel values of three-fourths of the patients with odonto type HPP were lower than the mean values of the healthy group. One-third of patients treated with enzyme replacement therapy showed higher corrected pixel values than the healthy group. Our results suggest that odonto type HPP without skeletal problems is occasionally accompanied by hypomineralization of alveolar bone and that alveolar bone hypomineralization in patients with severe HPP is possibly improved by enzyme replacement therapy.

## Introduction

Hypophosphatasia (HPP) is the inherited metabolic disease caused by mutations in the *ALPL* gene encoding tissue-nonspecific alkaline phosphatase (TNSALP) and is characterized by impaired mineralization of bones caused by low levels of alkaline phosphatase (ALP) activity^1–4^. Primary complications of HPP are bone mineral defects and premature loss of primary teeth (before the age of 4 years)^5^.

HPP is classified into six types by the age at onset and symptoms: perinatal, prenatal benign, infantile, childhood, adult, and odonto type HPP^1–5^. HPP was previously classified into five types without division of the perinatal type; however, prenatal benign HPP with a good life prognosis was then proposed^6^. Perinatal HPP is the most severe type with obvious symptoms at birth, whereas a child with infantile HPP presents with symptoms before 6 months of age. Patients with perinatal HPP and half of patients with infantile HPP have poor life prognoses without treatment because of respiratory complications^7^. In childhood type HPP, the first symptoms occur between 6 months and 18 years of age, whereas adult type HPP is diagnosed after the age of 18 years. Odonto type HPP is the mildest form, featuring dental complications at any age without bone symptoms. Early exfoliation of the primary incisors before 4 years of age sometimes leads to the diagnosis of mild HPP, such as childhood type HPP^8–13^. The clinical types of HPP are part of a continuous spectrum, and laboratory data overlap among the different forms^5^. Patients diagnosed in adulthood sometimes had HPP manifestations in childhood^14^. In a retrospective observational study of patients with adult type HPP, half of the patients had dental symptoms^15^. The types of HPP may change during the course of a patient’s life^2^. Odonto type HPP occasionally shifts to childhood or adult type HPP with bone symptoms as the patients age, and long-term follow-up of these children is mandatory^14–17^.

Enzyme replacement therapy (ERT) using bone-targeted recombinant ALP has improved the prognosis of patients with severe HPP^18–24^. The main dental symptom of HPP is early exfoliation of primary teeth caused by the hypomineralization of cementum^8–13^. Since the establishment of ERT in recent years, patients with severe HPP who did not survive until they were old enough to adapt to cooperative dental management are now able to visit dentists for dental treatment^25,26^. In addition to hypomineralization of cementum, many dental manifestations are detected as the patients age, such as hypomineralization of enamel and dentin, thin alveolar bone, malalignment, and occlusal problems^27^. However, the dental effects of ERT remain to be elucidated^27–29^.

**Figure 1:**
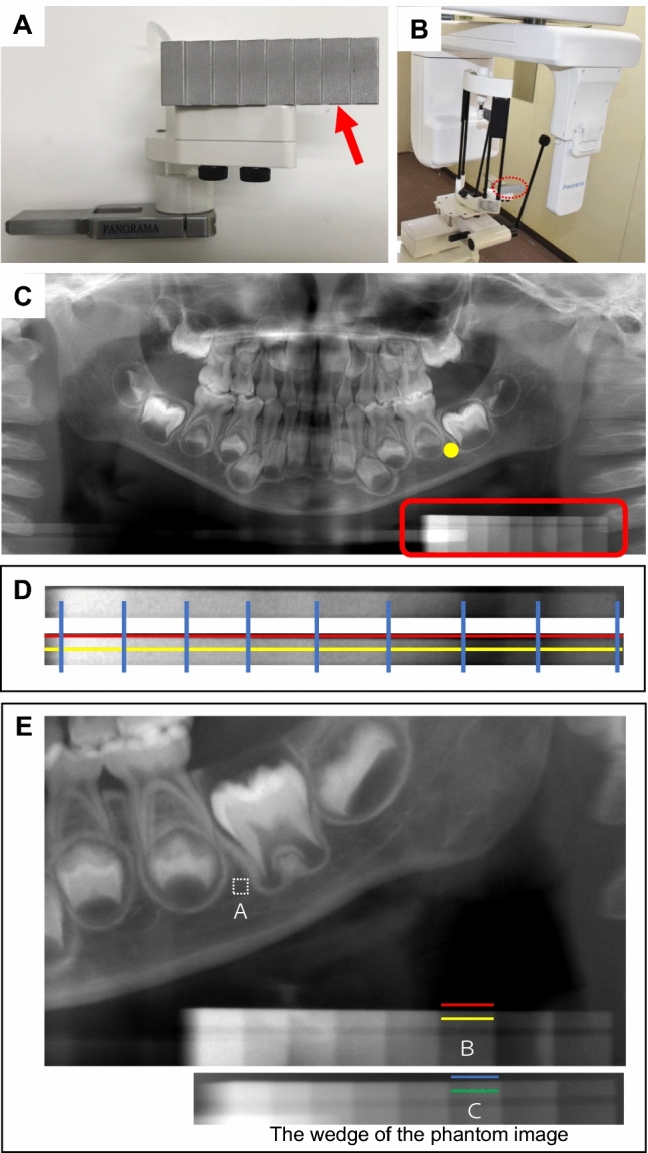
Methods for taking orthopantomographic images with the original indicator. (**A**) A step wedge (arrow) was fabricated for use as an indicator when applied to the chinrest of an orthopantomographic device. (**B**) The orthopantomographic image was taken using the special chinrest with the step wedge. The red dotted circle indicates the step wedge. (**C**) The orthopantomographic image was taken with the special chinrest. The step wedge was projected under the left side of the mandibular bone (red rectangle). The yellow dot on the orthopantomograph is the region of interest (ROI), which is a square area of 20 × 20 pixels. (**D**) Enlargement of the red square in (**C**), showing the wedge brightness correction. The blue vertical lines indicate the boundaries between the eight steps of wedge brightness for each orthopantomographic image. The yellow line shows the site at which brightness was measured inside the wedge of each step. The red line shows the site at which brightness was measured outside the wedge of each step. (**E**) If the average brightness of the ROI is (**A**), then (**B**) is the difference in average brightness between the yellow and red lines of the closest brightness step of the wedge on the same image. (**C**) Is the difference in average brightness between the green and blue lines at the same step of the wedge of the phantom image.

Orthopantomography, also called panoramic radiography, is a commonly used imaging modality in dental practice that serves as a valuable diagnostic tool^30^. Dentists can gain a full understanding of the dental symptoms in the entire tooth and oral maxillofacial region from orthopantomography^31,32^. This technique is also useful for observing the growth and development of the oral region over time, especially in pediatric dentistry^33,34^. Figure 1B,C show an example of an orthopantomographic imaging device and orthopantomographic image, respectively.

No standard index is currently available for evaluating alveolar bone hypomineralization in patients with HPP. The aim of this study was to establish a quantitative method for evaluating alveolar bone hypomineralization using orthopantomography, allowing comparison between the values of patients with HPP and the standard values of healthy subjects.

## Results

### Background of subjects

Orthopantomographic images were obtained from 200 healthy subjects (110 boys, 90 girls) and 17 patients with HPP (8 boys, 9 girls) from our clinic (Supplementary Table 1). Table 1 summarizes the medical details of the patients with HPP in the present study. The severity of the HPP in the patient population spanned from perinatal to odontohypophosphatasia. There were no significant differences between the average age at onset for patients with odonto type (range, 15–40 months) and childhood type (range, 9–21 months). There were also no significant differences between the average age at diagnosis for patients with odonto type (range, 21–71 months) and childhood type (range, 26–76 months). One patient with infantile type and two patients with childhood type were initially diagnosed based on the dental manifestation of early exfoliation of primary teeth. The mean serum ALP value was significantly higher in patients with odonto type than in those with perinatal type (*P* = 0.0016) and prenatal benign type (*P* = 0.0198). There was no significant difference in the mean serum ALP value of patients with odonto type and childhood type or between patients with odonto type and infantile type. All patients with perinatal and infantile types were receiving ERT. Information regarding the *ALPL* mutations in 15 patients showed that autosomal recessive inheritance was significantly more frequent in the non-odonto types than in the odonto type (*P* = 0.007).

**Table 1:** Characteristics of HPP patients.

HPP form	Perinatal	Prenatal benign	Infantile	Childhood	Odonto	Total
Total no. of patients (rate for all patients)	3 (18%)	2 (12%)	1 (6%)	3 (18%)	8 (47%)	17 (100%)
Age at onset Mean ± SEM (months) [median]	0.0 [0.0]	0.0 [0.0]	0.0 [0.0]	15.6 ± 3.7 [18.0]	25.7 ± 2.8 [22.8]	14.8 ± 3.2 [18.0]
Ages at diagnosis Mean ± SEM (months) [median]	0.0 [0.0]	0.0 [0.0]	19.2 [19.2]	42.4 ± 16.6 [26.4]	41.0 ± 5.5 [37.8]	27.9 ± 5.9 [26.4]
ALP value (U/L) Mean ± SEM	12.7 ± 6.7	52.0 ± 13.0	167.0	199.3 ± 86.3	278.8 ± 18.1	184.5 ± 30.9
Total no. of patients who received ERT (rate for all patients in each phenotype)	3 (100%)	1 (50%)	1 (100%)	1 (33%)	0 (0%)	6 (35%)
Age at start of ERT administration Mean ± SEM (months) [median]	0.0 [0.0]	0.0 [0.0]	20.4 [20.4]	142.8 [142.8]	–	27.2 ± 23.4 [0]
Duration of ERT administration Mean ± SEM (months) [median]	47.7 ± 9.2 [40.0]	41.0 [41.0]	14.6 [14.6]	34.2 [34.2]	–	38.8 ± 6.7 [38.5]
Inheritance patterns AR/AD	3/0	2/0	1/0	1/2	0/6 NA 2	7/8 NA 2

*ALP* alkaline phosphatase, *ERT* enzyme replacement therapy, *HPP* hypophosphatasia, *AR* autosomal recessive, *AD* autosomal dominant, *NA* not available, *SEM* standard error of the mean.

### Reproducibility of adjusted pixel value

A step wedge was fabricated for use as an indicator when applied to the chinrest of the orthopantomographic device and was placed to create vertical height below the inferior edge of the mandible during orthopantomography (Fig. 1). Reproducibility was checked 10 times with phantom scans on different days. The pixel values of each step wedge and the right and left region of interest (ROI) were calculated and adjusted (Supplementary Table 2). The reproducibility determined with the variation coefficient of the 10 phantom scans ranged from 1.7 to 3.9%, suggesting that the present method has high reproducibility.

### Corrected pixel value in healthy subjects

**Figure 2:**
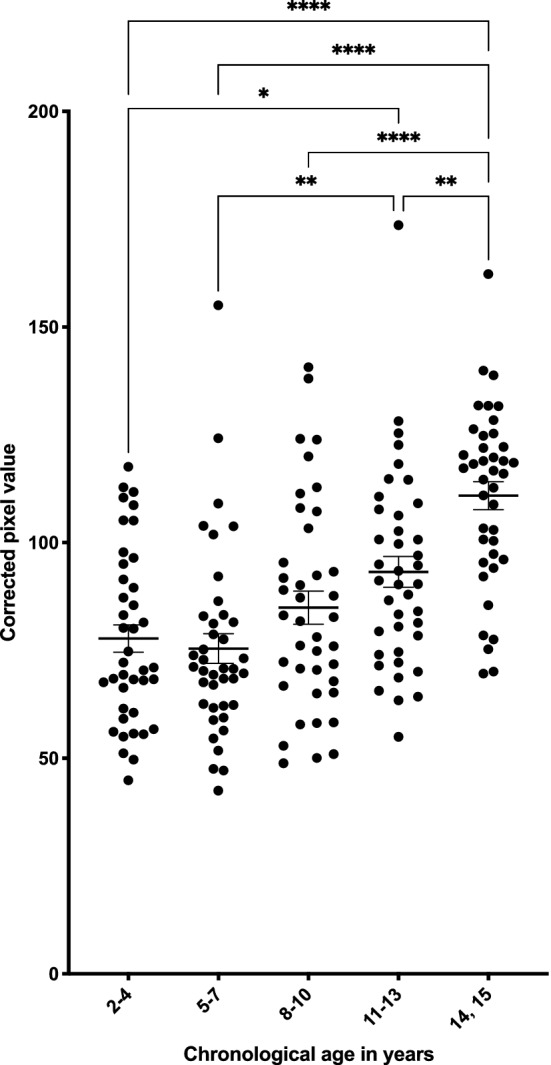
Distribution of corrected pixel values for each age group in years. Significant differences were determined using analysis of variance with Bonferroni correction. **P < 0.01 and ****P < 0.0001 versus 14–15 year group.

The corrected pixel values in the healthy subjects were 77.8 ± 4.5 (average ± SEM) in the 2–4 year group (n = 40), 75.5 ± 4.7 in the 5–7 year group (n = 40), 84.9 ± 4.9 in the 8–10 year group (n = 40), 93.2 ± 4.8 in the 11–13 year group (n = 40), and 110.9 ± 4.5 in the 14–15 year group (n = 40) (Fig. 2). Among the healthy subjects, the corrected pixel values of the 2–4, 5–7, 8–10, and 11–13 year groups were significantly lower than that of the 14–15 year group (2–4, 5–7, and 8–10 year groups versus 14–15 year group: *P* < 0.0001, 11–13 year group versus 14–15 year group: *P* = 0.0038) and increased with age.

### Chronological age, dental age, and corrected pixel values in healthy subjects

**Figure 3:**
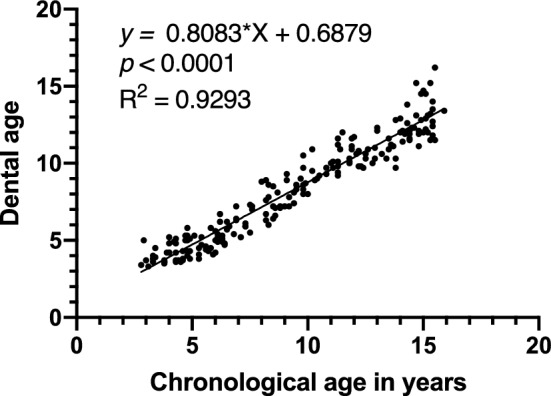
Correlation of chronological age and dental age. A significant positive correlation was found between chronological age and dental age (correlation coefficient = 0.9640).

**Figure 4:**
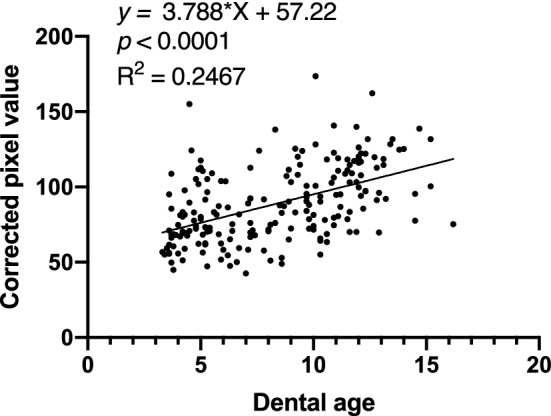
Correlation of the corrected pixel value and dental age. A positive correlation was found between the corrected pixel value and dental age (correlation coefficient = 0.4968).

Dental ages were evaluated in the orthopantomographic images according to the stages of the permanent teeth. However, the crown of the maxillary central incisor was not formed completely in one subject, and the tooth germ of the third molars could not be observed in another subject, demonstrating the difficulty in determining the dental age. These two subjects were therefore excluded from the analyses in the present study. A significant positive correlation was found between chronological age and dental age in the healthy subjects (*P* < 0.0001) (Fig. 3). Additionally, dental age was demonstrated to be positively and significantly correlated with the corrected pixel values in healthy subjects (*P* < 0.0001) (Fig. 4).

### Corrected pixel values and dental age in patients with HPP

**Figure 5:**
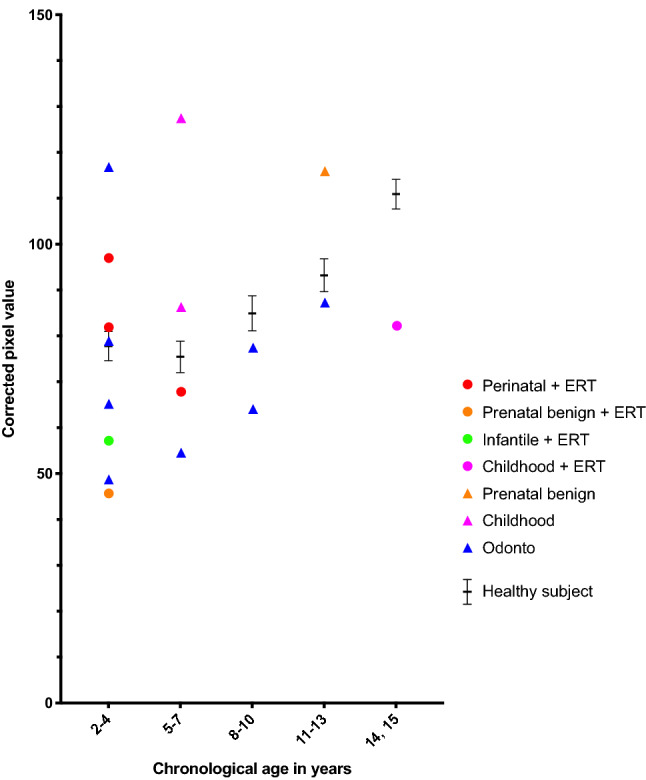
Comparison of the corrected pixel value of patients with each phenotype of hypophosphatasia with or without enzyme replacement therapy (ERT) and in healthy subjects. Circles represent treated groups and triangles represent untreated groups. Black horizontal lines shown in each chronological age group indicate average ± SEM of the healthy subjects.

The corrected pixel values of two patients with odonto type HPP aged 2–4 years, one patient with odonto type HPP aged 5–7 years, two patients with odonto type HPP aged 8–10 years, and one patient with odonto type HPP aged 11–13 years were lower than the mean values of the healthy subjects (Fig. 5). However, the corrected pixel values of all patients with HPP not receiving ERT were higher than the mean values of the healthy subjects. Additionally, the corrected pixel values of one-third of the patients with HPP receiving with ERT were higher than those of the healthy subject group of the same age.

### Correlation between chronological and dental age in patients with HPP

**Figure 6:**
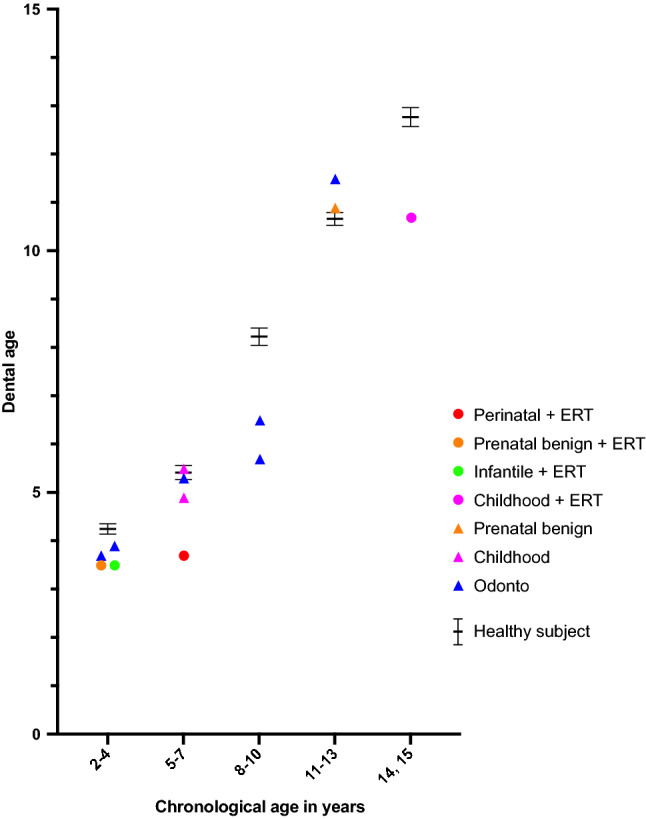
Comparison of dental age of patients with each phenotype of hypophosphatasia with or without enzyme replacement therapy (ERT) and in healthy subjects. Circles represent treated groups and triangles represent untreated groups. Black horizontal lines shown in each chronological age group indicate average ± SEM of the healthy subjects.

**Figure 7:**
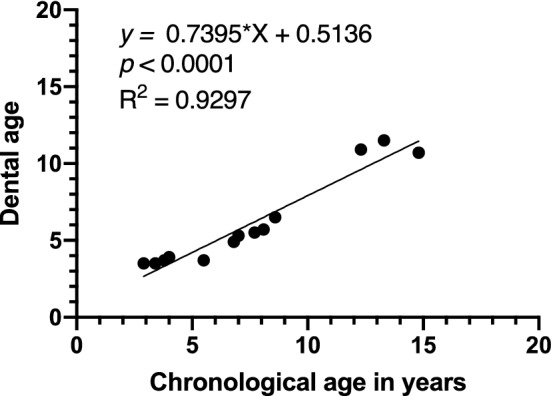
Correlation of chronological age and dental age. A significant positive correlation was found between chronological age and dental age in patients with hypophosphatasia (correlation coefficient = 0.9642).

The dental age of four patients with HPP could not be evaluated because the crown of the maxillary central incisor was not completely formed. The dental age of all patients with HPP treated with ERT was lower than the mean age of the healthy subjects (Fig. 6). A significant positive correlation was found between chronological and dental age in the patients with HPP (*P* < 0.0001) (Fig. 7). However, the gradient of the linear equation of patients with HPP was smaller than that of the healthy subjects, suggesting that patients with HPP had a lower dental age than that of the healthy subjects.

## Discussion

This is the first study to devise a method for quantitatively evaluating alveolar bone mineralization in orthopantomographic images of patients with HPP. A novel method was devised to quantitatively evaluate pixel values in orthopantomographic images using the step wedge as a reference. The pixel value of an orthopantomographic image is affected not only by the condition of the hard and soft tissues of an individual but also by the physique and position of the individual. To correct for these effects, we attempted to correct the pixel value of each image based on the step wedge by recording the step wedge at the same position for each examination. This is our original method and had not been previously used. By defining this corrected pixel value, it is possible to quantify the radiolucency and radiopacity of the orthopantomographic image. Different quantitative and qualitative indices calculated on orthopantomography have been proposed to screen for reduced skeletal bone mineral density (BMD) in osteoporosis^35,36^. BMD was predicted by quantitative analysis of the trabecular pattern on dental radiographs by Geraets et al*.*^35^. A systematic review of the linear and quantitative orthopantomographic measures to assess the accuracy of these indices was performed by Calciolari et al*.*^36^. They described limitations related to differences in the experience and agreement between different operators and the different image quality and magnification of the orthopantomographs. They also concluded that standardized orthopantomography and controlling for magnification and distortion are needed in detecting reduced skeletal bone density. The indicator we developed solves the problem of orthopantomography not having quantitative gray values like those of multidetector computed tomography images by attaching a simple indicator to the chinrest.

The corrected pixel values in the healthy subjects were significantly lower in the younger group than in those aged 14–15 years and increased with age. We chose the distal side of the mandibular left second premolar tooth germ in the primary or mixed dentition or the root apex of the second premolar in the permanent dentition as the measurement point for quantification of the corrected pixel values. Stable values were obtained in this study because this region is minimally affected by changes in the dentition. Several studies have evaluated BMD using mandibular cortical width in children with osteogenesis imperfecta, which is the most common skeletal disease reported in children^37,38^. Our method can be applied in future studies to measure the condition of the mandible in bone diseases that are accompanied by dental symptoms, such as osteogenesis imperfecta or X-linked hypophosphatemia.

Studies involving quantitative evaluations with orthopantomography for clinical osteoporosis screening have used the mandibular cortical index and the mandibular inferior cortical width below the mental foramen^39,40^. Evaluation of the alveolar trabecular bone pattern of the mandible using orthopantomography and intraoral radiography has also been reported^35^. Additionally, the density and fractal analysis of orthopantomography can be used to detect osteoporosis^41^. These studies used aluminum balls of different diameters to standardize brightness. Because these methods do not fully consider the change in brightness caused by overlapping soft tissues, we attempted to devise a modified quantitative evaluation method using orthopantomography.

A significant positive correlation was found between chronological age and dental age in healthy subjects. Dental age corresponds to the formation of the permanent tooth germs, which is an important index for assessing tooth mineralization. However, the actual dental age calculated in the present study should be regarded as lower than the chronological age in each case. This is because no data have been collected from Japanese children in recent times; therefore, we used data obtained from Scandinavian children 50 years ago^42^ when measuring dental age in our clinical practice. The gap between the chronological and dental ages in healthy subjects may have been a result of the use of dental ages of children of different races or eras. A positive correlation was also found between dental age and corrected pixel values in the healthy subjects, indicating that corrected pixel values increase with dental age because both the tooth and bone are calcified tissues.

Japanese patients with odonto type HPP, the mildest form of HPP with only dental symptoms, exhibit relatively higher serum ALP values than patients with the other types of HPP except for childhood type HPP, including perinatal, prenatal benign, and infantile type HPP^27^. Early exfoliation of primary teeth, which is the main dental manifestation, is caused by hypomineralization of cementum^8,9,12^. HPP is a clinically stable but chronic disorder in many affected children and adolescents^3^; however, it is a progressive disease in some patients, with the appearance of bone symptoms as patients age^2,16,17,27^.

The SD scores of the corrected pixel values of patients with odonto type HPP were within 2SD (Supplementary Table 3). This is consistent with the absence of bone symptoms in patients with odonto type HPP. However, the corrected pixel values of three-fourths of the patients with odonto type HPP were lower than the mean values of the healthy subjects in this study, suggesting that early exfoliation of primary teeth could be caused not only by hypomineralization of cementum but also by hypomineralization of alveolar bone. This finding also demonstrates that the reduction in serum ALP activity is associated with the mandibular bone density in patients with odonto type HPP who do not have skeletal bone symptoms or signs. Taken together, these findings indicate that the lower corrected pixel values in patients with odonto type HPP are a possible indicator of disease progression to the whole body. Additionally, the values may be a criterion for estimating the prognosis of odonto type HPP.

One-third of the patients with HPP treated with ERT had higher corrected pixel values than the healthy subject group of the same age. The serum ALP value at birth in patients with perinatal type HPP is almost 0, and such patients cannot survive without treatment because of respiratory failure caused by severe bone hypomineralization^7^. Their corrected pixel values were close to those of the healthy subjects in the present study, indicating that ERT reduces hypomineralization in mandibular bone to the same extent as in skeletal bone.

This is the first reported application of dental age as an indicator of dental symptoms of HPP. Our findings indicate that dental age is useful to evaluate tooth hypomineralization. The dental ages of patients with HPP were lower than those of the healthy subjects, indicating that tooth development in patients with HPP may be slower than that in healthy children. To our knowledge, no studies have investigated tooth formation speed as a dental manifestation of HPP in human subjects. We previously reported in a nationwide survey that hypomineralization of enamel and dentin was detected in patients with HPP, especially in those with severe HPP^27^. Delayed tooth formation is a novel dental finding in the patients with HPP observed in the present study. We consider that low serum ALP values influence not only bone hypomineralization but also tooth hypomineralization in patients with HPP. The dental age of all patients with HPP treated with ERT was lower than the mean dental age of the healthy subjects, suggesting that ERT may not reduce tooth hypomineralization to the same extent as skeletal bone hypomineralization. A limitation of this study is that it is a cross-sectional study of a rare disease. A longitudinal study of patients with HPP is necessary to study the effect of ERT in the oral region, and more cases should be included to confirm any statistically significant differences.

A major limitation of the study is the small number of patients with HPP because of the rarity of this disease. Only 17 patients with HPP were included in this study, which was not sufficient for statistical analysis. However, compared with the healthy subject groups of the same age, the corrected pixel values of three-fourths of the patients with odonto type HPP were lower and those of one-third of the patients with HPP treated with ERT were higher. These tendencies may be proved to be statistically significant with the accumulation of many more cases in the future.

The estimated frequency of severe HPP, mostly the perinatal and infantile types, is 1 per 150,000 in Japan and North America and 1 per 300,000 in European countries^43–46^. In the present study, patients with perinatal HPP had extremely low single-digit or double-digit ALP values, and all patients with perinatal and infantile HPP received ERT. ERT is an absolute indication if the patient is certain to have HPP and is predicted to have a poor prognosis, such as patients with the perinatal or infantile type^5,20^. However, the estimated frequency of heterozygous HPP is 1 per 6370 in European countries^4,47^. In the present study, patients with odonto type HPP comprised half of the total patients and had mostly dominant inheritance. It is estimated that many patients with odonto type HPP remain undiagnosed. There was no significant difference in the serum ALP values at diagnosis between patients with odonto type and childhood type HPP in this study. Additionally, dental manifestations were the first symptoms of HPP in three patients with infantile and childhood type HPP in this study. HPP is known to be a potentially progressive disease. Patients diagnosed with odonto type HPP with only dental manifestations occasionally transition to childhood type or adult type HPP with bone symptoms as they age^2,16,17,27^. Early diagnosis and management of growth and development are important for patients with HPP. Dentists are in a position to make an early diagnosis based on early exfoliation of the primary incisors.

In this study, we targeted subjects under the age of 16 years who had complete permanent dentition. However, the prognosis of severe HPP is greatly improved by ERT, and oral management is important for patients’ quality of life. Severe HPP is sometimes accompanied by severe dental problems^25–27^. Quantitative evaluation methods are needed to properly diagnose dental symptoms when patients may receive orthodontic treatment and implants in the future. Studies of adults are also needed because the end point of the standard values cannot be detected from this study.

We devised an innovative method of using orthopantomography for quantitative assessment of alveolar bone mineralization. This method revealed that odonto type HPP is sometimes accompanied by hypomineralization of the alveolar bone and teeth and that ERT is effective in reducing hypomineralization of the alveolar bone in patients with HPP.

## Methods

### Medical details of patients with HPP

The following patient details were assessed and recorded: chronological age at the time of orthopantomography, sex, phenotype (perinatal, prenatal benign, infantile, childhood, odonto), age at onset of HPP, age at diagnosis, serum ALP value (Japanese Society of Clinical Chemistry method^48^ at diagnosis), treatment with or without ERT, and type of *ALPL* mutation.

### Collection of orthopantomographic images

Healthy patients who visited our clinic for oral management were invited to participate in the study when they required orthopantomography as part of their treatment for dental caries, periodontal disease, or occlusal problems. Informed consent was obtained from all patients’ parents or legal guardians. The healthy subjects (aged 2–15 years) were classified into five age groups (2–4, 5–7, 8–10, 11–13, and 14–15 years). After informed consent had been obtained from 40 subjects per group, orthopantomographic images were taken with a special step wedge constructed for the present study. Images were obtained from the 200 healthy subjects. However, 16 subjects were excluded because radiolucent or radiopaque periapical lesions were observed near the ROI on the orthopantomographic images. Sixteen more healthy subjects were then recruited. Seventeen patients with HPP aged 2–15 years who visited our clinic for oral management were also invited to participate in this study.

### Orthopantomography

A step wedge was fabricated for use as an indicator when applied to the chinrest of the orthopantomographic device (Hyper-X; Asahi Roentgen Industries Co., Ltd., Kyoto, Japan) (Fig. 1A,B). Orthopantomographic images were taken with a tube current of 12 mA and tube voltage of 60 kV.

### Alveolar bone density

MATLAB 2017a in combination with the Image Processing Toolbox (MathWorks, Inc. Natick, MA, USA) was used for alveolar bone density analysis on DICOM (Digital Imaging and COmmunications in Medicine) data of orthopantomographic images. The ROI was manually placed at the distal side of the second mandibular premolar root or the primary second mandibular molar (Fig. 1C). The size of the ROI was 20 × 20 pixels. The average pixel value of this ROI was measured. If radiolucent or radiopaque lesions were recognized in this area, the patient’s data were excluded.

### Wedge brightness correction

The brightness of the mandible and the wedge on orthopantomographic images varies depending on the imaging model and the subject. Therefore, it is necessary to correct for differences in brightness as much as possible. We determined the boundary between eight steps of wedge brightness on each orthopantomographic image (blue vertical lines) (Fig. 1D). We measured the brightness inside the wedge of each step (yellow line, measured as close to the upper edge of the wedge as possible). We also measured the brightness of the background immediately outside the wedge of each step (red line). Finally, we subtracted the measured value of the red line from the measured value of the yellow line in each step. Thus, we determined the approximate value of the brightness of the wedge steps only, excluding the soft tissue brightness.

### ROI brightness correction

The difference in the brightness of the ROI also needed to be corrected on the orthopantomographic images. We therefore performed the following operations and calculations. Orthopantomographic images of a phantom were taken 10 times as a reference image using the same method as in Fig. 1, and the average brightness of the wedge was measured according to the method in Fig. 1D. A detailed explanation of the brightness correction is shown in Fig. 1E. The average brightness value inside the ROI was measured and defined as parameter A. The number of steps of the wedge on the same image closest to the average brightness of the ROI was determined. The average brightness of the step was defined as parameter B. The average brightness of the wedge of the phantom image at the same step was defined as parameter C. Parameters B and C are the brightness of the same wedge step and should be corrected to the same value. Thus, the ROI corrected brightness was calculated as follows:

$${\text{ROI correction density }} = {\text{ A }} - ({\text{B }} - {\text{ C}}).$$

To evaluate the reproducibility of the measurement, the corrected brightness values of the ROI of 10 orthopantomographic images of a phantom were calculated, and the coefficient of variation was measured (Supplementary Table 2). The coefficient of variation was 2.951% for the right ROI and 3.853% for the left ROI. Therefore, the measurement of the corrected brightness had sufficient reproducibility.

### Dental age

Dental age is estimated from the analysis of tooth maturation, wherein maturation is assessed by the occurrence of an event or a sequence of irreversible events that are subsequently compared with normal standards. Dental development is characterized by several structural changes throughout life, and these changes are effective physiological growth indicators. Teeth are less affected by environmental and nutritional changes than is bone, making teeth extremely useful in estimating age^49^. In the present study, dental ages were calculated using the method described by Haavikko^42^, which has been shown to be valid for application in Japanese subjects^50^. Haavikko reported age medians in years for 12 tooth formation stages for boys and girls separately as well as for the maxilla and mandible. These 12 tooth formation stages are divided into 12 stages that span from the period before the onset of calcification through crown formation to root closure. One pediatric dentist assessed the formation stages of all permanent teeth from the orthopantomographic images. The formation stages were converted to chronological age, and the average of those chronological ages was the dental age of the individual.

### Statistical analysis

Statistical analysis was performed using GraphPad Prism 9 (GraphPad Software Inc., La Jolla, CA, USA). Intergroup differences were compared using analysis of variance. Bonferroni correction was used for post-hoc analysis. Differences with a *P* value of < 0.05 were considered statistically significant. Differences in inheritance patterns were assessed by Fisher’s exact test. Pearson’s correlation analysis was performed to find the correlation between chronological age and dental age and between pixel value and dental age.

### Ethical approval

This study was conducted in full adherence to the Declaration of Helsinki (64th World Medical Association General Assembly, Fortaleza, Brazil, 2013) and the Ethical Guidelines for Medical and Health Research Involving Human Subjects. The study protocol was approved by the Ethics Committee of Osaka University Graduate School of Dentistry (approval no. H29-E26). Other institutions were approved by their own ethics committee as participating facilities based on our approval. All volunteers and patients were informed in writing and gave written informed consent to participate. All data were fully anonymized before they were accessed in this study. This trial was registered (UMIN000033623).

## Supplementary Information

Supplementary Table 1.

Supplementary Table 2.

Supplementary Table 3.

## Data Availability

The original contributions presented in the study are included in the article; further inquiries can be directed to the corresponding author.
