# Supplementary material for: Evaluation of alveolar bone hypomineralization in pediatric hypophosphatasia using orthopantomography
Source: Sci Rep. 2022 Jan 24;12:1211. doi: 10.1038/s41598-022-05171-5 (PMC8786966; doi:10.1038/s41598-022-05171-5)
Supplement: Supplementary file 1 — Supplementary Table 1. [file 41598_2022_5171_MOESM1_ESM.docx]

**Evaluation of alveolar bone hypomineralization in pediatric hypophosphatasia using orthopantomography**

Rena Okawa, Takashi Nakamoto, Saaya Matayoshi, Kazuhiko Nakano, Naoya Kakimoto

Supplementary Table 1. Characteristics of HPP patients

| Case no. | Age  (years) | Sex | HPP form | Age at onset  (months) | Age at diagnosis  (months) | ALP (U/L) | Age at start of ERT administration  (months) | Mutations of *ALPL* |
| --- | --- | --- | --- | --- | --- | --- | --- | --- |
| 1 | 14.8 | M | C | 20.4 | 26.4 | 66 | 142.8 | p.K224E/p.P409A |
| 2 | 13.3 | F | O | 39.6 | 51.6 | 255 | - | p.R71C heterozygous |
| 3 | 3.1 | M | P | 0.0 | 0 | 11 | 0.0 | p.Leu520RfsX86  /p.Y479C |
| 4 | 5.5 | F | P | 0.0 | 0 | 2 | 0.0 | p.G491R/p.G491R |
| 5 | 6.8 | F | C | 8.4 | 75.6 | 171 | - | p.R184W heterozygous |
| 6 | 8.1 | F | O | 30.0 | 42 | 254 | - | NA |
| 7 | 3.8 | F | O | 21.6 | 46.8 | 289 | - | p.E452K heterozygous |
| 8 | 8.6 | M | O | 14.4 | 20.4 | 192 | - | p.R184W heterozygous |
| 9 | 7.0 | M | O | 24.0 | 70.8 | 361 | - | p.R71C heterozygous |
| 10 | 7.7 | M | C | 18.0 | 25.2 | 361 | - | p.Leu520RfsX86  heterozygous |
| 11 | 12.3 | F | PB | 0.0 | 0 | 39 | - | p.Leu520RfsX86  /p.M226I |
| 12 | 4.0 | M | O | 21.6 | 30 | 289 | - | p.R184W heterozygous |
| 13 | 2.7 | M | O | 21.6 | 32.4 | 328 | - | p.H171R heterozygous |
| 14 | 2.8 | F | O | 32.4 | 33.6 | 262 | - | NA |
| 15 | 3.3 | F | P | 0.0 | 0 | 25 | 0.0 | p.Leu520RfsX86  /p.S188P |
| 16 | 3.4 | F | PB | 0.0 | 0 | 65 | 0.0 | p.F327L/ p.Leu520RfsX86 |
| 17 | 2.9 | M | I | 0.0 | 19.2 | 167 | 20.4 | p.H482Q/C/ p.Leu520RfsX86 |

ALP, alkaline phosphatase; C, childhood; ERT, enzyme replacement therapy; F, female; HPP, hypophosphatasia; I, infantile; M, male; NA, not available; O, odonto; P, perinatal; PB, prenatal benign.
