# Supplementary material for: Evaluation of alveolar bone hypomineralization in pediatric hypophosphatasia using orthopantomography
Source: Sci Rep. 2022 Jan 24;12:1211. doi: 10.1038/s41598-022-05171-5 (PMC8786966; doi:10.1038/s41598-022-05171-5)
Supplement: Supplementary file 2 — Supplementary Table 2. [file 41598_2022_5171_MOESM2_ESM.docx]

**E** **Evaluation of alveolar bone hypomineralization in pediatric hypophosphatasia using orthopantomography**

Rena Okawa, Takashi Nakamoto, Saaya Matayoshi, Kazuhiko Nakano, Naoya Kakimoto

Supplementary Table 2. Reproduction of adjusted pixel values of the region of interest (ROI)

|  | Pixel value of each step wedge | | | | | | | | Right ROI | Adjusted right ROI | Left ROI | Adjusted left ROI |
| --- | --- | --- | --- | --- | --- | --- | --- | --- | --- | --- | --- | --- |
|  | 1st | 2nd | 3rd | 4th | 5th | 6th | 7th | 8th |  |  |  |  |
| Base | 123.8729 | 106.0968 | 86.4143 | 68.22 | 54.3974 | 43.7059 | 31.8681 | 17.4579 | 109.9850 | - | 105.8125 | - |
| Day 1 | 122.0877 | 102.3175 | 85.2803 | 67.6806 | 53.8734 | 41.9583 | 30.0989 | 16.3404 | 111.0725 | 114.8518 | 105.6700 | 109.4493 |
| Day 2 | 123.1083 | 108.7419 | 88.4783 | 70.6164 | 54.1076 | 40.1687 | 26.9556 | 13.0266 | 111.0225 | 108.3774 | 104.4800 | 101.8349 |
| Day 3 | 122.6119 | 103.2417 | 81.2817 | 63.0694 | 48.6914 | 37.9277 | 27.7611 | 15.1737 | 112.3850 | 115.2401 | 104.1250 | 106.9801 |
| Day 4 | 120.6462 | 104.4127 | 84.7353 | 68.1867 | 54.0000 | 41.9819 | 30.1720 | 15.0000 | 108.5000 | 110.1841 | 107.9725 | 109.6566 |
| Day 5 | 124.4444 | 107.0238 | 86.0882 | 68.9648 | 55.1090 | 41.1024 | 29.0225 | 14.9053 | 110.3875 | 109.4605 | 103.0600 | 102.1330 |
| Day 6 | 120.3780 | 103.4462 | 86.7456 | 71.2973 | 55.7200 | 43.2346 | 28.5966 | 14.1889 | 107.3775 | 110.0281 | 109.5625 | 112.2131 |
| Day 7 | 125.5411 | 109.2154 | 88.2721 | 71.6944 | 56.2961 | 42.8605 | 30.7360 | 16.9202 | 112.2200 | 109.1014 | 104.1009 | 100.9823 |
| Day 8 | 123.8468 | 109.3906 | 91.4714 | 74.5845 | 58.9313 | 43.5843 | 29.6722 | 14.2473 | 107.4825 | 104.1887 | 108.4275 | 105.1337 |
| Day 9 | 125.6786 | 110.3529 | 91.6484 | 73.5753 | 58.2564 | 44.5610 | 30.9176 | 16.3474 | 109.6275 | 107.8218 | 104.4425 | 102.6368 |
| Day 10 | 122.0610 | 109.0469 | 87.8529 | 70.8592 | 56.5938 | 42.4107 | 29.8791 | 16.1882 | 111.6775 | 108.7274 | 104.5850 | 101.6349 |
| Average | | | | | | | | | 110.17530 | 109.79813 | 105.64260 | 105.26547 |
| Standard deviation | | | | | | | | | 1.85812 | 3.24024 | 2.20632 | 4.0556 |
| Coefficient of variation | | | | | | | | | 1.687 | 2.951 | 2.088 | 3.853 |
