# Supplementary material for: Evaluation of alveolar bone hypomineralization in pediatric hypophosphatasia using orthopantomography
Source: Sci Rep. 2022 Jan 24;12:1211. doi: 10.1038/s41598-022-05171-5 (PMC8786966; doi:10.1038/s41598-022-05171-5)
Supplement: Supplementary file 3 — Supplementary Table 3. [file 41598_2022_5171_MOESM3_ESM.docx]

**Evaluation of alveolar bone hypomineralization in pediatric hypophosphatasia using orthopantomography**

Rena Okawa, Takashi Nakamoto, Saaya Matayoshi, Kazuhiko Nakano, Naoya Kakimoto

Supplementary Table 3. Corrected pixel values of patients with odonto type hypophosphatasia

| Case no. | Age (years) | Corrected pixel value | SD score |
| --- | --- | --- | --- |
| 2 | 13.3 | 87.6 | −0.25 |
| 6 | 8.1 | 64.4 | −0.85 |
| 7 | 3.8 | 117.1 | 1.96 |
| 8 | 8.6 | 77.8 | −0.29 |
| 9 | 7.0 | 55.0 | −0.95 |
| 12 | 4.0 | 65.6 | −0.61 |
| 13 | 2.7 | 49.1 | −1.43 |
| 14 | 2.8 | 79.3 | 0.07 |
